# Effect of Chain Polydispersity on the Elasticity of Disordered Polymer Networks

**DOI:** 10.1021/acs.macromol.1c00176

**Published:** 2021-04-14

**Authors:** Valerio Sorichetti, Andrea Ninarello, José M. Ruiz-Franco, Virginie Hugouvieux, Walter Kob, Emanuela Zaccarelli, Lorenzo Rovigatti

**Affiliations:** † Laboratoire de Physique Théorique et Modéles Statistiques (LPTMS), CNRS, Université Paris-Saclay, F-91405 Orsay, France; ‡ Laboratoire Charles Coulomb (L2C), University of Montpellier, CNRS, F-34095 Montpellier, France; § IATE, University of Montpellier, INRAE, Institut Agro, F-34060 Montpellier, France; ∥ CNR-ISC Uos Sapienza, Piazzale A. Moro 2, IT-00185 Roma, Italy; ⊥ Department of Physics, Sapienza Università di Roma, Piazzale A. Moro 2, IT-00185 Roma, Italy; # Institut Universitaire de France, 75005 Paris, France

## Abstract

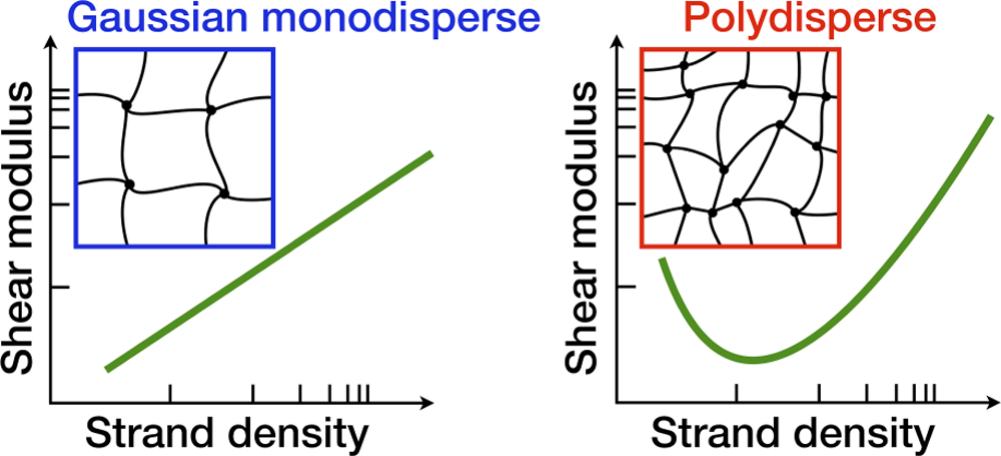

Due to their unique structural and mechanical properties, randomly cross-linked polymer networks play an important role in many different fields, ranging from cellular biology to industrial processes. In order to elucidate how these properties are controlled by the physical details of the network (e.g., chain-length and end-to-end distributions), we generate disordered phantom networks with different cross-linker concentrations *C* and initial densities ρ_init_ and evaluate their elastic properties. We find that the shear modulus computed at the same strand concentration for networks with the same *C*, which determines the number of chains and the chain-length distribution, depends strongly on the preparation protocol of the network, here controlled by ρ_init_. We rationalize this dependence by employing a generic stress–strain relation for polymer networks that does not rely on the specific form of the polymer end-to-end distance distribution. We find that the shear modulus of the networks is a nonmonotonic function of the density of elastically active strands, and that this behavior has a purely entropic origin. Our results show that if short chains are abundant, as it is always the case for randomly cross-linked polymer networks, the knowledge of the exact chain conformation distribution is essential for correctly predicting the elastic properties. Finally, we apply our theoretical approach to literature experimental data, qualitatively confirming our interpretations.

## 1 Introduction

For many applications, the elasticity of a cross-linked polymer network is one of its most important macroscopic properties.^1^ It is thus not surprising that a lot of effort has been devoted to understanding how the features of a network, such as the fraction and functionality of cross-linkers or the details of the microscopic interactions between chain segments, contribute to generate its elastic response.^2−6^ The macroscopic behavior of a real polymer network (be it a rubber or a hydrogel) depends on many quantities, such as the properties of the polymer and of the solvent, the synthesis protocol, and the thermodynamic parameters. However, in experiments, it is difficult to disentangle how these different elements contribute to the elastic properties of the material. This task becomes easier in simulations because all the relevant parameters can be controlled in detail.^7−16^ In this regard, an important feature of real polymer networks that can be exploited is that their elasticity can be described approximately as the sum of two contributions: one due to the cross-linkers and one due to the entanglements.^15−18^ The former can be approximated well by the elastic contribution of the corresponding phantom network,^19^ that is, when the excluded volume between the strands is not taken into account.^15,16^ It is, therefore, very important to understand the role that the chain conformation distribution plays in determining the dynamics and elasticity of phantom polymer models.

The distribution of the chemical lengths of the strands connecting any two cross-linkers in a network, that is, the chains (chain-length distribution for short), depends on the chemical details and on the synthesis protocol. In randomly cross-linked networks, this distribution is typically exponential,^7,20^ whereas monodisperse or quasimonodisperse networks can be obtained by using specific end-linking protocols, for example, via the assembly of tetra-PEG macromers with a small polydispersity.^21^ Regardless of the synthesis route, the presence of short or stretched chains is common, although the exact form of the chain conformation fluctuations is highly nontrivial. From a theoretical viewpoint, however, the majority of the results on the elasticity of polymer networks have been obtained within the mean-field realm, in which scaling assumptions and chain Gaussianity are assumed.^19,22^ Therefore, simulations can be extremely helpful to clarify the exact role played by the chain-length distribution and better understand the experimental results. However, most simulation studies have focused on melt densities, where random or end-cross-linking can be carried out efficiently,^7−10,12,14−16^ or have employed idealized lattice networks.^11,13,23−25^ This makes it challenging to compare the results from such simulations with common experimental systems such as hydrogels, which are both low-density and disordered.^26^

In the present paper, we show that the knowledge of the exact chain end-to-end distribution is essential to correctly predict the linear elastic response of low-density polymer networks. We do so by simulating disordered phantom networks generated with different cross-linker concentrations *C* and initial monomer densities ρ_init_. In our systems, the former parameter controls the number of chains and the chain-length distribution, while the latter determines the initial end-to-end distance distribution of the chains and, therefore, plays a similar role as the solvent quality in an experimental synthesis. To generate the gels, we exploit a recently introduced technique based on the self-assembly of patchy particles, which has been found to to correctly reproduce structural properties of experimental microgels.^27−29^ This method allows us to obtain systems at densities comparable with those of experimental hydrogels, that is, giving access to swelling regimes inaccessible through the previously employed techniques based on numerical vulcanization of high-density polymer melts.^8−13,15,16^ We first demonstrate that systems generated with the same *C* but at different values of ρ_init_ can display very different elastic properties even when probed at the same strand concentration, despite having the same chain length distribution. Second, we compare the numerical results to the phantom network theory.^19^ In order to do so, we determine the theoretical relation between the shear modulus *G* and the single-chain entropy for generic non-Gaussian chains. We find a good agreement between theory and simulation only for the case in which the exact chain end-to-end distribution is given as an input to the theory, with some quantitative deviations appearing at low densities. On the other hand, assuming a Gaussian behavior of the chains leads to qualitatively wrong predictions for all the investigated systems except the highest density ones. Overall, our analysis shows that for low-density polymer networks and in the presence of short chains, the knowledge of the exact chain conformational fluctuations is crucial to predict the system elastic properties reliably. Notably, we validate our approach against recently published experimental data,^21,30^ showing that the behavior of systems where short chains are present cannot be modeled without precise knowledge of the chain-size-dependent end-to-end distribution.

## 2 Theoretical Background

In this section, we review some theoretical results on the elasticity of polymer networks, for the most part available in the literature,^1,19,22^ by reorganizing them and introducing the terminology and notation that will be employed in the rest of the paper. We will consider a polydisperse polymer network made of cross-linkers of valence ϕ connected by *N*_s_ strands. Here and in the following, we will assume the network to be composed of *N*_s_ elastically active strands, defined as strands with the two ends connected to distinct cross-linkers, that is, that are neither dangling ends nor closed loops (i.e., loops of order one). Moreover, for those strands, which are part of higher-order loops, we assume their elasticity to be independent of the loop order (see Zhong et al.^31^ and Lin et al.^32^). We will focus on evaluating the shear modulus *G* of the gel, which relates a pure shear strain to the corresponding stress in the linear elastic regime.^33^ One can theoretically compute *G* by considering uniaxial deformations of strain λ along, for instance, the *x* axis. We assume the system to be isotropic; moreover, since we are interested in systems with no excluded volume interactions, we assume a volume-preserving transformation,^a^ that is, λ_*x*_ = λ and λ_*y*_ = λ_*z*_ = λ^–1/2^ as extents of deformation along the three axes.

**Figure 1:**
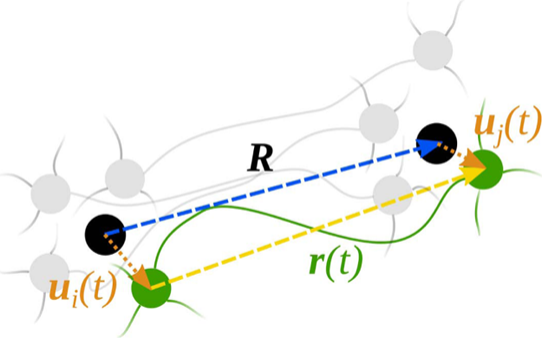
Cartoon providing a visual explanation of some of the quantities used throughout the text. In the cartoon, the black dots are the average positions of two cross-linkers *i* and *j*, the green dots are their instantaneous positions at time *t*, and the gray dots are their positions at some other times. The time-averaged end-to-end vector **R** is the vector connecting the black dots, the instantaneous end-to-end vector **r**(*t*) is the vector connecting the green dots, and its instantaneous fluctuation is the difference between the instantaneous fluctuations of the positions of the two cross-linkers, **u**_*i*_(*t*) and **u**_*j*_(*t*).

The starting point to calculate the shear modulus is the single-chain entropy, which is a function of the chain’s end-to-end distance.^22^ In general, we can write the instantaneous end-to-end vector of a single chain, which connects any two cross-linkers as **r**(*t*) = **R** + **u**(*t*), where  represents the time-averaged end-to-end vector and **u**(*t*) the fluctuation term (see Figure 1 for a cartoon depicting these quantities). We also assume that there are no excluded volume interactions, so that the chains can freely cross each other. We thus have ,^b^ because , the position and fluctuations of cross-linkers being uncorrelated.^19^

The entropy of a chain with end-to-end vector **r** = (*r*_*x*_, *r*_*y*_, *r*_*z*_) is *S*_n_(**r**) = *k*_B_ log  *W*_n_(**r**) + *A*_n_,^34^ where *W*_n_(**r**) is the end-to-end probability density of **r** and *A*_n_ is a temperature-dependent parameter that can be set to zero in this context. If the three spatial directions are independent (which is the case, e.g., if *W*_n_(**r**) is Gaussian) then *W*_n_(**r**) can be written as the product of three functions of *r*_*x*_, *r*_*y*_, and *r*_*z*_, so that *S*_n_(**r**) = *s*_n_(*r*_*x*_) + *s*_n_(*r*_*y*_) + *s*_n_(*r*_*z*_), where *s*_n_ is the entropy of a one-dimensional chain. Building upon this result, we can assume that each chain in the network can be replaced by three independent one-dimensional chains parallel to the axes using the so-called three-chain approximation.^1,35^ This assumption is exact for Gaussian chains, although for non-Gaussian chains the associated error is small if the strain is not too large.^1^

We will also assume (i) that the length of each chain in the unstrained state (λ = 1) is , and (ii) that, upon deformation, the chains deform affinely with the network, so that the length of the chain oriented along the *x* axis becomes *r̃*_λ_ and those of the chains oriented along the *y* and *z* axes become *r*_λ^–1/2^_. With those assumptions, the single-chain entropy *S*_n_(λ) becomes^1^
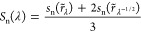
1where we need to divide by three because we are replacing each unstrained chain with end-to-end distance *r̃* by three fictitious chains of the same size. Usually, the λ-dependence of *r̃*_λ_ is controlled by the microscopic model and by the macroscopic conditions (density, temperature, etc.). Two well-known limiting cases are the affine network model,^19^ in which both the average positions and fluctuations of the cross-linkers deform affinely, *r̃*_λ_ = λ*r̃*, and the phantom network model,^19^ in which the fluctuations are independent of the extent of the deformation, so that
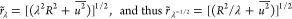
2

The free-energy difference between the deformed and undeformed state of a generic chain is Δ*F* = −*T*[*S*_n_(λ) – *S*_n_(1)], and thus the *x* component of the tensile force is given by
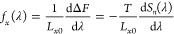
3

The latter quantity divided by the section *L*_*y*0_*L*_*z*0_ yields the *xx* component of the stress tensor, which thus reads
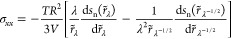
4where we have used eq 2.

Because the volume is kept constant, the Poisson ratio is 1/2^33^ and hence the single-chain shear modulus *g* is connected to the Young’s modulus  by *g* = *Y*/3, which implies that
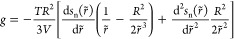
5We note that, although similar equations can be found in Smith^35^ and Treloar,^1^ to the best of our knowledge eq 5 has not been reported in the literature in this form. In order to obtain the total shear modulus *G* of the network, and under the assumption that the effect of higher-order loops can be neglected,^31,32^ one has to sum over the *N*_s_ elastically active chains. Of course, the result will depend on the specific form chosen for the entropy *s*_n_. We stress that a closed-form expression of the end-to-end probability density *W*_n_(**r**) is not needed because only its derivatives play a role in the calculation. Hence, it is sufficient to know the force–extension relation for the chain, because, as discussed above, the component of the force along the pulling direction satisfies eq 3 (see also Appendix A).

For a freely jointed chain (FJC)^22^ of *n* bonds of length *b*, *W*_n_(**r**) has the following form^1,36^
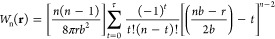
6where τ = ⌊(*nb* – *r*)/2*b*⌋, that is, the largest integer smaller than (*nb* – *r*)/2*b*.

In the limit of large *n*, eq 6 reduces to a Gaussian^36^
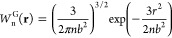
7

Under this approximation, the shear modulus takes the well-known form
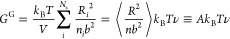
8where ν = *N*_s_/*V* is the number density of elastically active strands and *A* is often called the front factor.^35,37−39^ We have also introduced the notation ⟨·⟩ = *N*_s_^–1^∑_*i*_^*N*_s_^· for the average over all the strands in the system. In the particular case that the  values of the different chains are Gaussian-distributed (a distinct assumption from the one that *W*_n_(**r**) is Gaussian), which is the case, for example, for end-cross-linking starting from a melt of precursor chains,^10^ it can be shown that  (we recall that ϕ is the cross-linker valence), so that one obtains the commonly reported expression (see also Appendix A)^19,22^
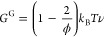
9Equation 8 was derived from eq 5, which assumes the validity of the phantom network model. If one assumes, on the other hand, that the affine network model is valid, a different expression for *G* is obtained (see Supporting Information).

To obtain a more accurate description of the end-to-end probability distribution for strained polymer networks, one has to go beyond the Gaussian model and introduce more refined theoretical assumptions. Among other approaches, the Langevin-FJC^1^ (L-FJC), the extensible-FJC^40^ (ex-FJC), and the worm-like chain^41^ (WLC) have been extensively used in the literature. In the L-FJC model, the force–extension relation is approximated using an inverse Langevin function, whereas in the ex-FJC model bonds are modeled as harmonic springs. These models give a better description of the system’s elasticity when large deformations are considered. The WLC model, in which chains are represented as continuously flexible rods, is useful when modeling polymers with a high persistence length (compared to the Kuhn length). More details about these models can be found in Appendix A.

## 3 Models and Methods

We build the polymer networks by employing the method reported in Gnan et al.,^27^ which makes use of the self-assembly of a binary mixture of limited-valence particles. Particles of species A can form up to four bonds (valence ϕ = 4) and bond only to B particles, thus acting as cross-linkers. Particles of species B can form up to two bonds (ϕ = 2) and can bond to A and B particles. We carry out the assembly of *N*_init_ = *N*_A_ + *N*_B_ = 5 × 10^4^ particles at different number densities ρ_init_ = *N*_init_/*V*, with *V* the volume of the simulation box, and different cross-linker concentrations *C* = *N*_A_/(*N*_B_ + *N*_A_). We consider two initial densities ρ_init_ = 0.1, 0.85, and *C* = 1, 5, and 10%. The results are averaged over two system realizations for each pair of ρ_init_, *C* values.

The assembly proceeds until an almost fully bonded percolating network is attained, that is, the fraction of formed bonds is at least *N*_bond_/*N*_bond_^max^ = 99.9%, where *N*_bond_^max^=(4*N*_A_ + 2*N*_B_)/2 is the maximum number of bonds. The self-assembly process is greatly accelerated thanks to an efficient bond-swapping mechanism.^42^ When the desired fraction *N*_bond_/*N*_bond_^max^ is reached, we stop the assembly, identify the percolating network, and remove all particles or clusters that do not belong to it. Because some particles are removed, at the end of the procedure the values of ρ_init_ and *C* change slightly. However, these changes are small (at most 10%) and in the following we will hence use the nominal (initial) values of ρ_init_ and *C* to refer to the different networks.

**Figure 2:**
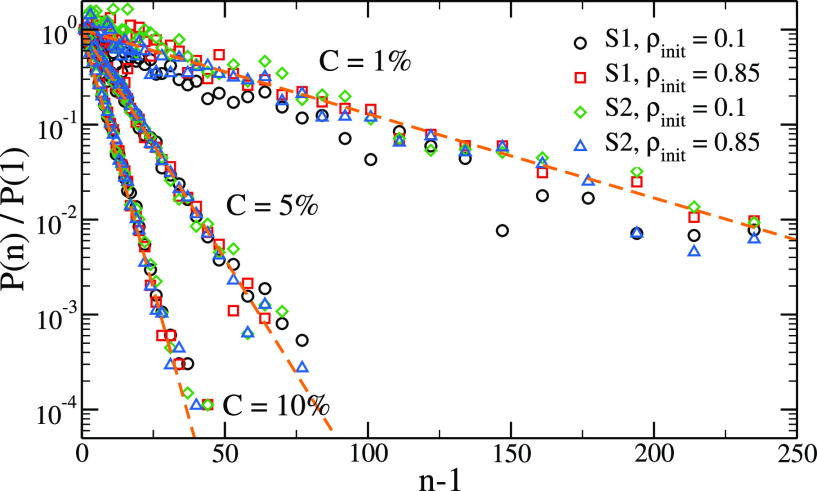
Rescaled distribution of chain lengths for all the simulated systems. We report the data for two samples (S1 and S2) generated with two values of initial density each. The orange dashed lines are the theoretical prediction of eq 10.

The normalized distribution of the chemical lengths *n* of the chains, *P*(*n*)/*P*(1), which constitute the network is shown in Figure 2. Here, the chemical chain length is defined as the number of particles in a chain, excluding the cross-linkers, so that a chain with *n* + 1 bonds has length *n*.^c^ In all cases, the distribution decays exponentially, as it is also the case for random-cross-linking from a melt of precursor chains.^7^ This exponential behavior can be explained by the Flory–Huggins polymerization theory^43^ or, equivalently, by the Wertheim theory for associating fluids applied to patchy particles,^44^ and it is ultimately due to the independence of the bonding events. We also note that *P*(*n*) does not depend on the initial density^27,45^ and, as one expects given the equilibrium nature of the assembly protocol, it is fully reproducible. This distribution can be estimated from the nominal values of ϕ and *C* via the well-known formula of Flory^43^
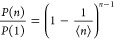
10where ⟨*n*⟩ = 2(1 – *C*)/ϕ*C* is the mean chain length,^45^ which, using the nominal cross-linker valence (ϕ = 4) and concentrations, takes the values 49.5, 9.5, and 4.5 for *C* = 1, 5, and 10%, respectively. The parameter-free theoretical probability distribution is shown as orange-dashed lines in Figure 2 and reproduces almost perfectly the numerical data.

The network contains a few defects in the form of dangling ends (chains that are connected to the percolating network by one cross-linker only) and first-order loops, that is, chains having both ends connected to the same cross-linker.^32^ Because there are no excluded volume interactions, these defects are elastically inactive and, therefore, do not influence the elastic properties of the network.^32,46,47^ For the configurations assembled at *C* = 1%, the percentage of particles belonging to the dangling ends is ≈10% for ρ_init_ = 0.1 and ≈6% for ρ_init_ = 0.85. For higher values of *C*, the percentages are much smaller (e.g., ≈ 2% for *C* = 5%, ρ_init_ = 0.1 and ≈1% for *C* = 10%, ρ_init_ = 0.1). In order to obtain an ideal fully bonded network, the dangling ends are removed. We note that during this procedure, the cross-linkers connected to dangling ends have their valence reduced from ϕ = 4 to ϕ = 3 or 2 (in the latter case, they become type B particles). The percentage of the so-created three-valent cross-linkers remains small: for ρ_init_ = 0.1, it is ≈15, 4, and 2% for *C* = 1, 5, and 10%, respectively. The presence of these cross-linkers slightly changes the average cross-linker valence, but does not influence the main results of this work.

Once the network is formed, we change the interaction potential, making the bonds permanent and thus fixing the topology of the network. Since we are interested in understanding the roles that topology and chain size distribution of a polymer network play in determining its elasticity, we consider interactions only between bonded neighbors, similar to what has been done in Duering et al.^10^ Particles that do not share a bond do not feel any mutual interaction, and hence chains can freely cross each other (whence the name phantom network). Two bonded particles interact through the widely used Kremer–Grest potential,^48^ which is given by the sum of a repulsive term accounting for steric interactions, modeled via the Weeks–Chandler–Andersen potential,^49^ and of a finite extensible nonlinear elastic (FENE) potential modeling the intramolecular bonds. The former is given by
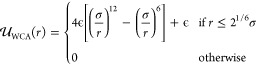
11where σ is the monomer diameter and ϵ sets the scale of the repulsion, while the latter is
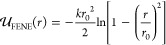
12where *k* = 30ϵ/σ^2^ and *r*_0_ = 1.5σ. Here and in the following, all quantities are given in reduced units: the units of energy, length, and mass are, respectively, ϵ, σ, and *m*, where ϵ and σ are defined by eq 11 and *m* is the mass of a particle, which is the same for A and B particles. The units of temperature, density, time, and elastic moduli are, respectively, [T] = ϵ/*k*_B_, [ρ] = σ^–3^, , and [G] = ϵσ^–3^. In these units, the Kuhn length of the model is *b* = 0.97.^48^

We run molecular dynamics simulations in the *NVT* ensemble at constant temperature *T* = 1.0 by employing a Nosé–Hoover thermostat.^50^ Simulations are carried out using the LAMMPS simulation package,^51^ with a simulation time step δ*t* = 0.003.

In order to study the effects of the density on the elastic properties, the initial configurations are slowly and isotropically compressed or expanded to reach the target densities ρ = 0.1, 0.2, 0.5, 0.85, and 1.5. Then, a short annealing of 10^6^ steps and subsequently a production run of 10^7^ steps are carried out. Even for the system with the longest chains, the mean-squared displacement of the single particles reaches a plateau, indicating that the chains have equilibrated (see the Supporting Information).

For each final density value, we run several simulations for which we perform a uniaxial deformation in the range λ_α_ ∈ [0.8, 1.2] along a direction α, where λ_α_ = *L*_α_/*L*_α,0_ is the extent of the deformation and *L*_α,0_ and *L*_α_ are the initial and final box lengths along α, respectively. The deformation is carried out at constant volume with a deformation rate of 10^–1^. To confirm that the system is isotropic, we perform the deformation along different spatial directions α.

**Figure 3:**
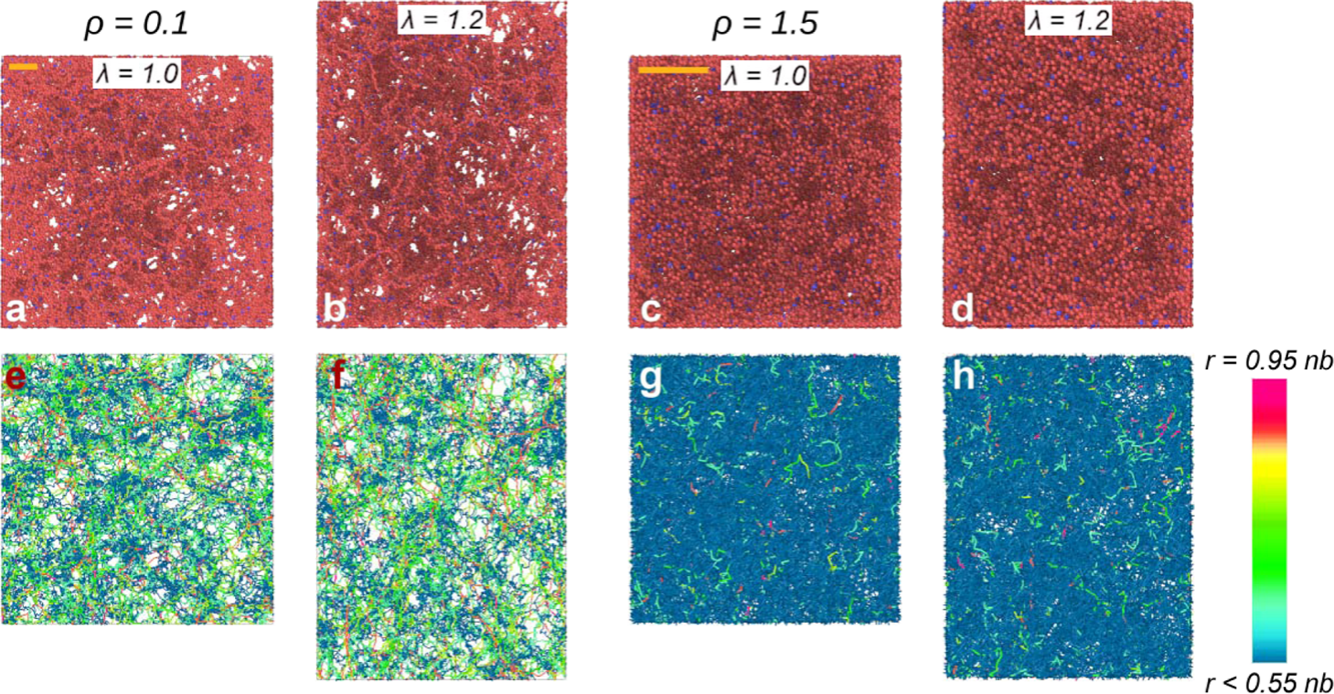
Snapshots of a network with cross-linker concentration *C* = 5% and assembly density ρ_init_ = 0.1 simulated at (a, b, e, and f) ρ = 0.1 and (c, d, g, and h) ρ = 1.5. We show configurations in equilibrium (a, c, e, and g) and subject to a uniaxial deformation along the vertical direction with λ = 1.2 (b, d, f, and h). Top row (panels a–d): turquoise and red particles indicate cross-linkers and monomers, respectively. The orange scale bars in the top left corners are 10σ long. Bottom row (panels e–h): the same configurations are represented in a way such that the chains are colored according to the ratio between their end-to-end distance and contour length (see the legend on the right).

Figure 3 shows representative snapshots of the *C* = 5%, ρ_init_ = 0.1 system at low (ρ = 0.1) and high (ρ = 1.5) density, in equilibrium and subject to a uniaxial deformation along the vertical direction. In panels 3a–d, we show the particles (monomers and cross-linkers), highlighting the highly disordered nature of the systems and their structural heterogeneity, which is especially evident at low density. The same systems are also shown in panels 3e–h, where we use a gradient to color chains according to the ratio between their end-to-end distance and contour length. These panels highlight the effect that density has on the heterogeneous elastic response of these systems when they are subject to deformations.

Once the system acquires the target value of λ_*z*_, we determine the diagonal elements of the stress tensor σ_αα_ and compute the engineering stress σ_eng_ as^22^
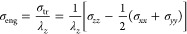
13where σ_tr_ is the so-called true stress.^1,52^ The shear modulus *G* is then the quantity that connects the engineering stress and the strain through the following relation^22^
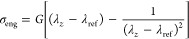
14In eq 14, λ_ref_ is an extra fit parameter that we add to take into account the fact that in some cases σ_eng_ ≠ 0 for λ_*z*_ = 1, which signals the presence of some prestrain in our configurations. The stress–strain curves we use to estimate *G* are averaged over 10 independent configurations obtained by the randomization of the particle velocities, prior to deformation, with a Gaussian distribution of mean value *T* = 1.0ϵ/*k*_B_ in order to reduce the statistical noise.

**Figure 4:**
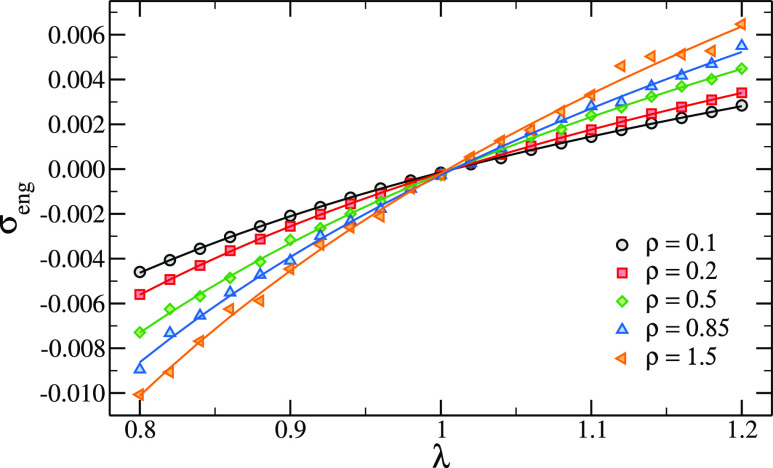
Example of stress–strain curves for the *C* = 1%, ρ_init_ = 0.1 system. Symbols are simulation data, lines are fits with eq 14.

Figure 4 shows the numerical data for the stress–strain curves for the *C* = 1%, ρ_init_ = 0.1 system. We also report the associated theoretical curves, fitted to eq 14, through which we obtain an estimate of the shear modulus.

## 4 Results and Discussion

We use the simulation data to estimate  (RMS end-to-end distance) and *R* ≡ *r̅* for each chain to compute the elastic moduli of the networks through eq 5. In the following, we will refer to the elastic moduli computed in this way with the term “theoretical”.

**Figure 5:**
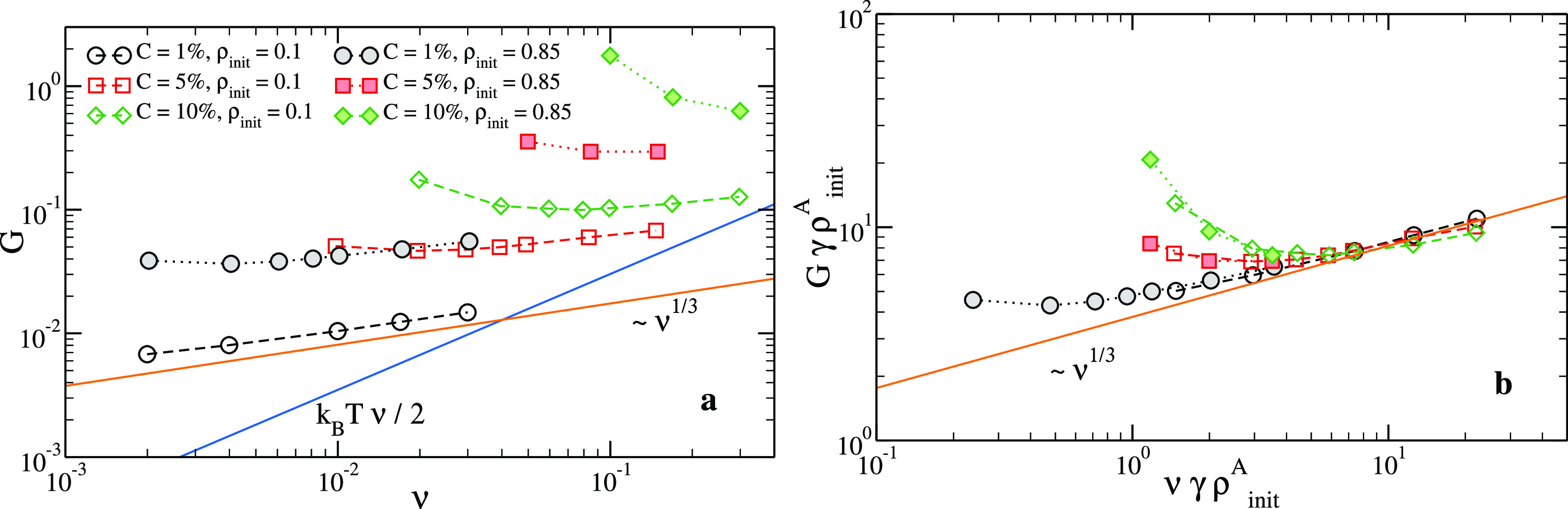
(a) Shear modulus as a function of the elastically active strand number density ν for all the investigated systems. Solid blue line: eq 9 with ϕ = 4. Solid orange line: slope 1/3. (b) Same as (a), with both *G* and ν rescaled by γρ_init_^A^, where γ = 0.74 for ρ_init_ = 0.85, γ = 1 for ρ_init_ = 0.1 is a fit parameter, and ρ_init_^A^ = =*C*ρ_init_ is the initial cross-linker density.

Figure 5a shows the shear modulus as computed in simulations for all investigated systems as a function of ν, the density of elastically active strands. We note that, to a first approximation, one would have ν = ϕ*C*ρ/2. However, the actual value of ν is slightly smaller than this figure because of the presence of elastically inactive strands, as discussed in Section 3. First of all, we observe that systems generated at the same *C* but with different values of ρ_init_ exhibit markedly different values of the shear modulus when probed under the same conditions (i.e., the same strand density). This result highlights the fundamental role of the cross-linking process, which greatly affects the initial distribution of the chains’ end-to-end distances even when the number of chains and their chemical length distribution, being dependent only on *C* (see Figure 2), are left unaltered. Thus, the echo of the difference between the initial end-to-end distributions gives rise to distinct elastic properties of the phantom networks even at the same strand density.

In Figure 5a, we also plot the behavior predicted by eq 9 (blue line), which assumes Gaussian-distributed end-to-end distances. Even though the numerical data seem to approach this limit at very large values of the density, they do so with a slope that is clearly smaller than unity. For the *C* = 1% sample, this slope is almost exactly 1/3, and it is also very close to this value for the *C* = 5% and *C* = 10% samples assembled at ρ_init_ = 0.1. This behavior can be understood at the qualitative level from eq 8: *R* is the average distance between cross-linkers and, therefore, it changes affinely upon compression or expansion, thereby scaling as *R* ∝ ν^–1/3^.^53,54^ As a result, in the Gaussian limit, the shear modulus scales as *G*^G^ ∝ ν^1/3^.^21,53−55^ As discussed above, our results show that the way this limiting regime is approached depends on the cross-linker concentration *C* and on the preparation state, which is here controlled by ρ_init_.

The quantitative differences in the elastic response of systems with different *C* and ρ_init_ can be partially rationalized by looking at the scaling properties of the end-to-end distances. We notice that the RMS equilibrium end-to-end distance *R*(*n*) of the strands for different values of ρ_init_ and *C* nearly collapses on a master curve when divided by the initial cross-linker density, ρ_init_^A^ = *C*ρ_init_ (see Supporting Information). A slightly better agreement is found if the heuristic factor γρ_init_^A^, with γ = 0.74 for ρ_init_ = 0.85 and γ = 1 for ρ_init_ = 0.1, is used. Based on this observation, we rescale the data of Figure 5a multiplying both *G* and ν by γρ_init_^A^. The result is shown in Figure 5b: one can see that the shear modulus of systems with the same *C* but different values of ρ_init_ nicely fall on the same curve. Moreover, in the large-ν limit, where all the curves tend to have the same slope, a good collapse of the data of systems with different *C* is also observed.

The differences arising between systems at different *C* can be explained by noting that the cross-linker concentration controls the relative abundance of chains with different *n*, whose elastic response cannot be rescaled on top of each other by using *n* but depends on their specific end-to-end distribution (see e.g., Appendix A). As a result, the elasticity of networks generated at different *C* cannot be rescaled on top of each other. In particular, systems with more cross-linkers, and hence more short chains, will deviate earlier and more strongly from the Gaussian behavior.

**Figure 6:**
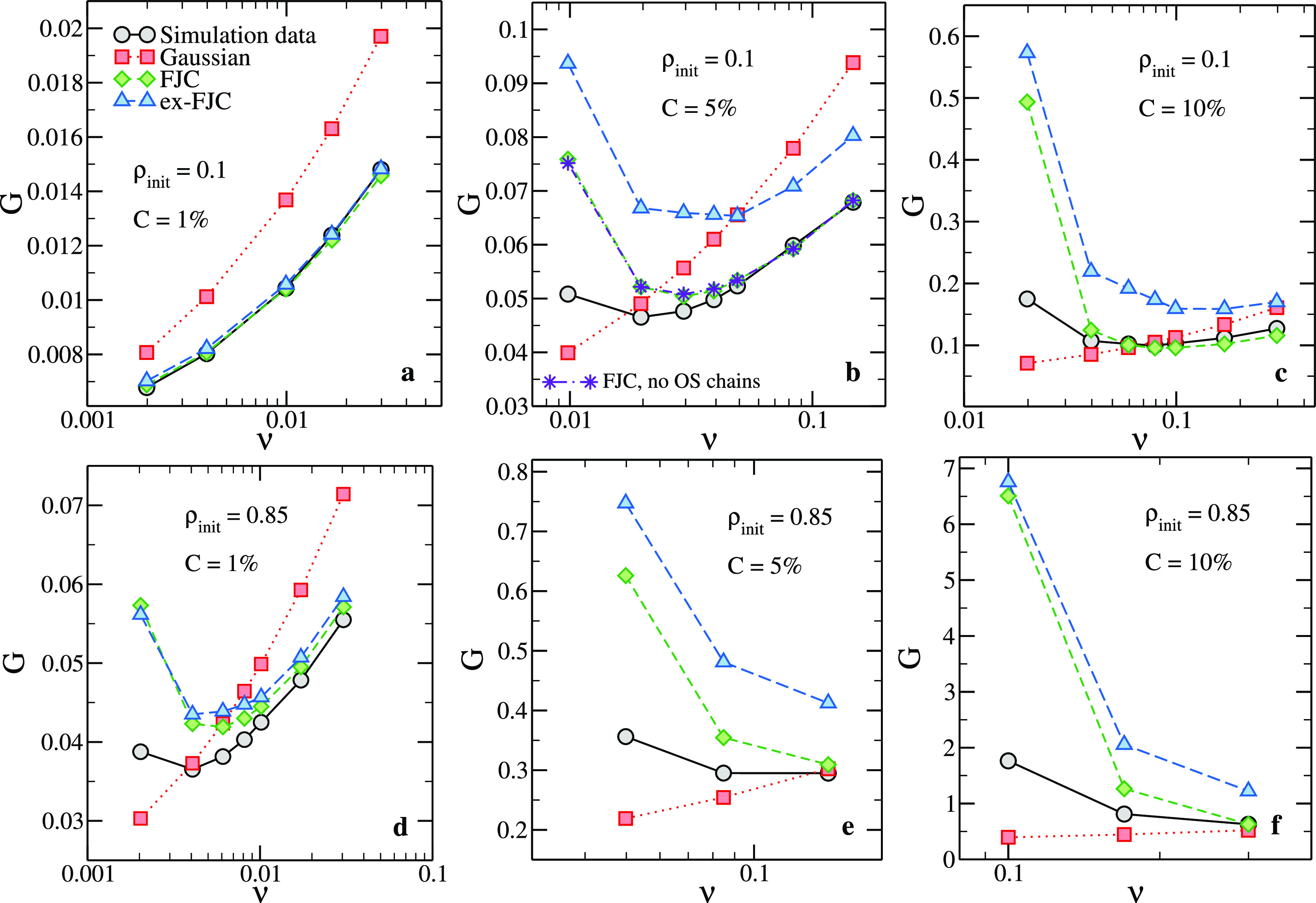
Comparison between the shear moduli obtained through eq 5 and three different approximations and the numerical ones (*G*) for the simulated systems (see legends). Dashed-dotted line/stars in panel b: FJC approximation with no overstretched chains (chains with *r* ≥ 0.95·*nb*).

Interestingly, *G* exhibits a nonmonotonic behavior as a function of ν; this feature appears for all but the lowest *C* and ρ_init_ values. This behavior, which has also been observed in hydrogels,^21,30,53,54,56^ cannot be explained assuming that the chains are Gaussian because in this case one has for all ν that *G* ∝ ν^1/3^, as discussed above. Given that our model features stretchable bonds, at large strains it cannot be considered to be a FJC, being more akin to an ex-FJC.^40^ Therefore, one might be tempted to ascribe the increase of *G* upon decreasing ν to the energetic contribution. For this reason, in addition to the Gaussian and FJC descriptions, we also plot in Figure 6b the shear modulus estimated by neglecting the contributions of those chains that have *r* ≥ 0.95·*nb*. We note that chains with *r* < 0.95·*nb* behave essentially as FJCs, and that in any case the number of such chains is minuscule in all but the lowest-density *C* = 10% systems (see the Supporting Information). Because the sets of data with and without the overstretched chains overlap almost perfectly, we confirm that the energetic contribution due to the few overstretched chains is negligible: we can thus conclude that the nonmonotonicity we observe has a purely entropic origin. This holds true for all the systems investigated except for the *C* = 10%, ρ_init_ = 0.85 system, which contains the largest number of short, overstretched chains (see Supporting Information).

In Figure 6, we compare the numerical shear modulus for all investigated systems with estimates as predicted by different theories, with the common assumption that the three-chain model remains valid (see Section 2). In particular, we show the results obtained with the FJC (eq 6), Gaussian (eq 7), and ex-FJC (see Appendix A) models. One can see that the agreement between the theoretical and numerical results is always better for larger values of ν, that is, when chains are less stretched. Moreover, the agreement between data and theory is better for systems generated at smaller ρ_init_. We note that the Gaussian approximation, which predicts a monotonically increasing dependence on ν, fails to reproduce the qualitative behavior of *G*, whereas the ex-FJC systematically overestimates *G*. The FJC description is the one that consistently achieves the best results, although it fails (dramatically at large *C*) at small densities. We ascribe this qualitative behavior to the progressive failure of the three-chain assumption as the density decreases. Because the three-chain model is known to overestimate the stress at large strains compared to more complex and realistic approximations such as the tetrahedral model,^1^ the resulting single-chain contribution to the elastic modulus for stretched chains is most likely overestimated as well. Regardless of the specific model used, our results suggest that when the samples are strongly swollen, something that can be achieved in experiments,^21^ any description that attempts to model the network as a set of independent chains gives rise to an unreliable estimate of the overall elasticity even when energetic contributions due to stretched bonds do not play a role.

In addition to providing the best comparison with the numerical data in the whole density range, the FJC description also captures the presence and (although only in a semiquantitative fashion) the position of the minimum. This is the case for all the investigated systems, highlighting the role played by the short chains, whose strong non-Gaussian character heavily influences the overall elasticity of the network.

**Figure 7:**
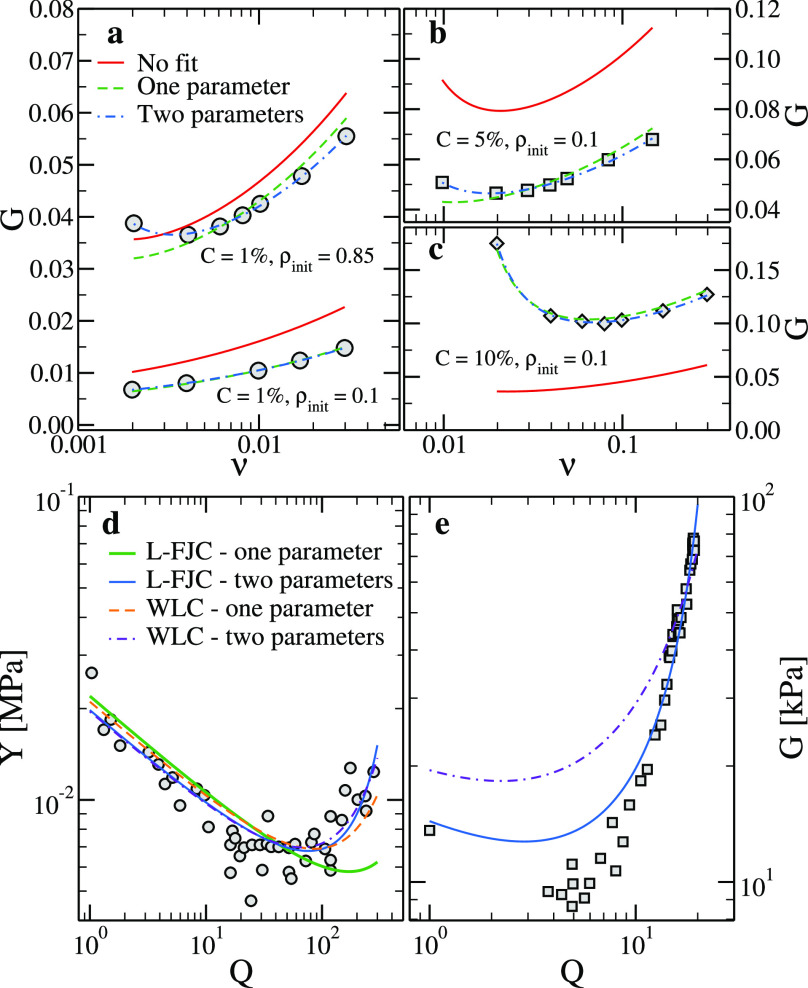
Fitting results to (a–c) simulation data (a) *C* = 1%, (b) *C* = 5%, and (c) *C* = 10% and (d,e) experimental data: (d) Young’s modulus taken from Hoshino et al.^21^ and (e) shear modulus taken from Matsuda et al.^30^ The quality of the fits in panel e does not depend on whether the point at *Q* = 1 is considered or not or if we restrict the fit to swelling ratios Q ≲ 15.

Although real short chains do not follow the exact end-to-end probability distribution we use here (see eq 6), they are surely far from the scaling regime and hence they should never be regarded as Gaussian chains, even in the melt or close to the theta point. This aspect has important consequences for the analysis of experimental randomly cross-linked polymer networks, for which one may attempt to extract some microscopic parameter (such as the contour length or the average end-to-end distance) by fitting the measured elastic properties to some theoretical relations such as the ones we discuss here. Unfortunately, such an approach will most likely yield unreliable estimates. We demonstrate that this happens even with a very idealized system such as the one we consider here. In order to follow the procedure usually applied to these polymeric systems,^21^ we make the assumption that the network can be considered as composed of *N*_s_ strands of ⟨*n*⟩ segments. We then compare in Figure 7a–c, the numerically estimated values of *G* with those obtained with the L-FJC model (see Appendix A). The expression we employ contains two quantities that can be either fixed or fitted to the data: the average end-to-end distance in a specific state (e.g., the preparation state), *R*_0_, and the average strand length ⟨*n*⟩ (or, equivalently, the contour length *r*_max_ = ⟨*n*⟩*b*). Together with the numerical data, in Figure 7 we present three sets of theoretical curves: *G* as estimated by using the simulation values of *R*_0_ and ⟨*n*⟩ or fitted by using either *R*_0_ or both quantities as free parameters. If *C* is small (and hence ⟨*n*⟩ is large), the difference between the parameter-free expression and the numerical data is small (10–15%). However, as *C* becomes comparable with the values that are often used in real randomly cross-linked hydrogels (≈5%), the difference between the theoretical and simulation data becomes very significant: for instance, for *C* = 10% the parameter-free expression fails to even capture the presence of the minimum. Fitting the numerical data makes it possible to achieve an excellent agreement, although the values of the parameters come out to be sensibly different (sometimes more than 50%) from the real values (see Appendix A). Our results thus show that even in the simplest randomly cross-linked system—a phantom network of freely jointed chains—neglecting the shortness of the majority of the chains, which dominate the elastic response, can lead to a dramatic loss of accuracy. Randomly cross-linked polymer networks contain short chains, which are inevitably quite far from the scaling regime, and hence even their qualitative behavior can become elusive if looked through the lens of polymer theories that rest too heavily on the Gaussianity of the chains.

To conclude our analysis, we also apply our theoretical expressions to two sets of data, which have been recently published. Both experiments have been carried out in the group of Gong.^21,30^ The first system is a tetra-PEG hydrogel composed of monodisperse long chains that can be greatly swollen by using a combined approach of adding a molecular stent and applying a PEG dehydration method.^21^ Because the system is monodisperse and the chains are quite long, we expect the theoretical expressions derived here to work well. Indeed, as shown in Figure 7d, the resulting Young’s modulus is a nonmonotonic function of the swelling ratio *Q*, defined as the ratio between the volume at which the measurements are performed and the volume at which the sample was synthesized. The experimental data can be fitted with both the L-FJC and WLC expressions (see Appendix A) because both models reproduce the data with high accuracy when fitted with the two free parameters introduced above (*R*_0_ and ⟨*n*⟩). However, better results are obtained with the WLC model, which fits well when ⟨*n*⟩ is fixed to its experimentally estimated value, yielding a value *R*_0_ = 7.2 nm, which is very close to the independently estimated value of 8.1 nm,^21^ in agreement with what reported in Hoshino et al.^21^

The second system we compare to is a randomly cross-linked PNaAMPS network, for which the shear modulus as a function of *Q* has been reported.^30^ As shown in Figure 7e, the theoretical expressions reported here cannot go beyond a qualitative agreement with the experimental data, even if two parameters are left free and we only fit to the experimental data in a narrow range of swelling ratios. In addition, the fitting procedure always yields unphysical values for the two parameters (e.g., *R*_0_ comes out to be smaller than 1 nm, see Appendix A). Although part of the discrepancy might be due to the charged nature of the polymers involved,^57^ we believe that the disagreement between the theoretical and experimental behaviors can be partially ascribed to the randomly cross-linked nature of the network, and hence to the abundance of short chains. Because the end-to-end distribution of such short chains is not known and depends on the chemical and physical details, there is no way of taking into account their contribution to the overall elasticity in a realistic way. These results thus highlight the difficulty of deriving a theoretical expression to assess the elastic behavior of randomly cross-linked real networks.

## 5 Summary and Conclusions

We have used numerical simulations of disordered phantom polymer networks to understand the role played by the chain size distribution in determining their elastic properties. In order to do so, we employed an in silico synthesis technique by means of which we can independently control the number and chemical size of the chains, set by the cross-linker concentration, as well as the distribution of their end-to-end distances, which can be controlled by varying the initial monomer concentration. We found that networks composed of chains of equal contour length can have shear moduli that depend strongly on the end-to-end distance even when probed at the same strand concentration. Hence, this shows that even in simple systems the synthesis protocol can have a large impact on the final material properties of the network even when it does not affect the chemical properties of its basic constituents, as recently highlighted in a microgel system.^58^

We then compared the results from the simulations of the phantom network polymer theory, which was revisited to obtain explicit expressions for the shear modulus assuming three different chain conformation fluctuations, namely, the exact freely jointed chain, Gaussian, and extensible freely jointed chain models. We observed a nonmonotonic behavior of *G* as a function of the strand density that, thanks to a comparison with the theoretical results, can be completely ascribed to entropic effects that cannot be accounted for within a Gaussian description. We thus conclude that the role played by short-stretched chains in the mechanical description of polymer networks is fundamental and should not be overlooked. This insight is supported by an analysis of experimental data of the elastic moduli of hydrogels reported in the literature. We are confident that the numerical and analytical tools employed here can be used to address similar and other open questions concerning both the dynamics and the topology in systems in which excluded-volume effects are also taken into account, and hence entanglements effects may be relevant. Investigations in this direction are underway.

## Figures and Tables

**Figure 8:**
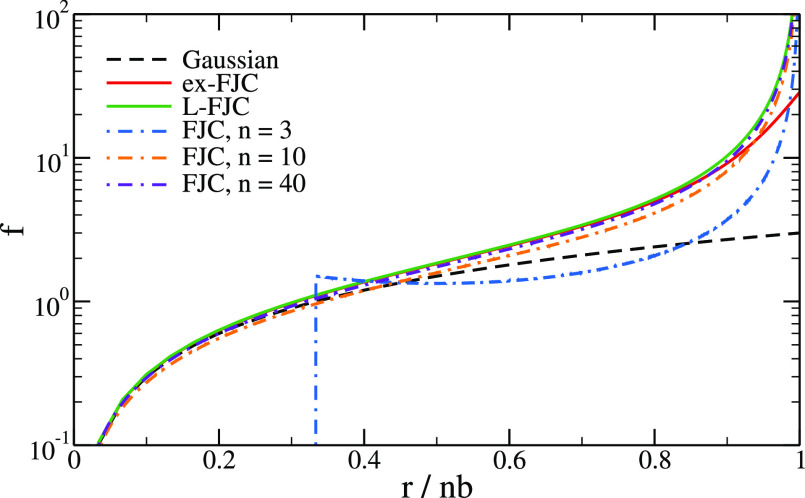
Force required to extend a chain by *r* (scaled by its contour length *nb*) for the Gaussian (dashed line, eq 7), L-FJC and ex-JFC (solid lines, eq 21), and FJC (dashed-dotted lines, eq 6) models at *T = 1*. Note that being plotted in this way, *f* depends on *n* only for the latter case.

## Appendix A

### Shear Modulus of a System with Gaussian-Distributed End-to-End Distances

In this section, we show how eq 9 can be derived for a polydisperse network. Similar derivations for the case of monodisperse networks can be found in standard textbooks.^19,22^ We start by noting something that is sometimes overlooked: the Gaussian distribution *W*_n_^G^(**r**), eq 7, applies to a single chain. However, at the ensemble level, the distribution of end-to-end vectors, which we may call Ω[**r**(*n*)], is no Gaussian in general. However, if we assume, for example, that the system has been obtained through end-cross-linking starting from a melt of precursor chains,^10^ then Ω[**r**(*n*)] = *W*_n_^G^(**r**),^34^ so that the magnitudes *r* of the **r** vectors will be Gaussian-distributed. Under this assumption, one has

15

To evaluate the term in brackets in eq 8, we thus only need to evaluate the fluctuation term . This term can be computed using the equipartition theorem (the same derivation can be found, e.g., in ref (59)). The total energy of the fluctuations is , where *N*_*x*_ is the number of active cross-linkers because there is one mode for each node and to each mode it is associated an energy of 3/2*k*_B_*T*.^59^ Moreover, *N*_*x*_ = 2*N*_s_/ϕ, with *N*_s_ the number of elastically active strands. Therefore, the mean energy per strand is

16

where the sum extends over all the elastically active strands. Therefore, we get  (a generalization to the polydisperse case of a well-known result for the phantom network^20,60^), from which we finally obtain

17

From eq 8, we obtain eq 9, that is, .

The validity of eq 17 depends not only on the cross-linking procedure but also on the macroscopic thermodynamic parameters (such as solvent quality, density, or pressure). For example, if the chain-size distribution is such that short chains are abundant, as it is the case for randomly cross-linked networks,^7,20^ the front factor A (see eq 8) will depend on the chain size distribution because short chains are non-Gaussian. Another example is when the cross-linking procedure is performed in a state in which the chains are non-Gaussian, for example, under good solvent conditions, where the chains behave as self-avoiding random walks.^22^

### Models of Chain Statistics

The freely jointed chain approximation, eq 6, describes an inextensible chain because each component *f*_α_, where α = *x*, *y*, and *z*, of the force **f** required to stretch the chain, for example

18

diverges in the limit *r* → *nb*, that is, when the end-to-end distance approaches the contour length. In the limit of large strains and large degree of polymerization *n*, a better approximation is provided by the well-known Langevin dependence of the elongation on the exerted force *f*, which yields for the end-to-end probability distribution function^1^

19

where β = 1/*k*_B_*T*, *T* is the temperature, *k*_B_ is the Boltzmann constant, *A* is a normalization constant, and  is the inverse Langevin function. The latter is defined as  and it turns out to be equal to the ratio between the end-to-end distance and the contour length of a chain that is subject to a force *f*

20

We note on passing that  cannot be written in a closed form^61^ and hence must be evaluated numerically.

Phantom Kremer–Grest chains behave exactly as freely jointed chains up to end-to-end distances that are very close to the contour length. However, beyond those values the Langevin description is no longer valid, and one has to resort to the ex-FJC model, for which the following analytical form of the force–extension curve has been recently derived^40^

21

where *k* is the monomer–monomer force constant in the harmonic approximation. In principle, it is possible to integrate the inverse of this relation to get the end-to-end probability density. However, as it is clear from eq 5, we only need the derivatives of the inverse function, and hence there is no need to obtain *W*(*r*) explicitly. We set the value of *k* to the value of the second derivative of the Kremer–Grest potential as computed in the minimum, *k* ≈ 867 ϵ/σ^2^. We have also fitted the force–extension curves as obtained in simulations of single chains, obtaining values that are compatible with this latter estimate.

We also report the following expression, which approximates the force–elongation relation for a WLC^42^ and is used in the main text to fit the experimental data shown in Figure 7c

22

Figure 8 shows the force–extension curve for polymer chains described with different models. Because the force is plotted as a function of the distance scaled by the FJC contour length *nb*, the Gaussian, L-FJC and ex-FJC descriptions become independent of *n*. By contrast, the extensional force of the exact FJC model, whose end-to-end probability distribution is given by eq 6, retains an *n*-dependence that is very strong for small *n* and decreases upon increasing the chain size. Indeed, for *n* ≳ 40, the resulting force is essentially *n*-independent and overlaps almost completely with the FJC curve.

### Fitting Procedure and Additional Results

As discussed in the main text in Section 4, we fit the experimental Young’s and shear moduli reported in Hoshino et al.^21^ (system A) and Matsuda et al.^30^ (system B). As commonly done in the analysis of experimental systems, in both cases, we will consider the networks to be formed by strands of average size ⟨*n*⟩ and use relations based on eq 5: for system A, we use

23

while for system B, we fit *Y*_exp_ = 3*G*_exp_. We fit the experimental data with the L-FJC and WLC models by using eqs 19 and 22 to numerically evaluate the derivatives of *s*_n_(*r**~*).

We evaluate *R*, *r**~*, and ν as follows. Let *V*_0_, ν_0_, and *R*_0_ be the volume, chain number density, and average end-to-end distance of the polymer chains in the preparation state, respectively, and *V*, ν, and *R* be the same quantities for the generic state point at which the experimental measurements are carried out. We define the swelling ratio *Q* = *V*/*V*_0_ = ν_0_/ν so that ν = ν_0_/*Q* and . Since  and, as shown in Appendix A, , for systems with ϕ = 4 we also have that , where *r*_max_ = ⟨*n*⟩*b* is the contour length of the strands. Using the numbers reported in the original papers, we found that ν_0_^A^ = 1.97 × 10^–3^ nm^–3^ and ν_0_^B^ = 0.245 nm^–3^. For system A, the authors also report independent estimates for *R*_0_ (*R*_0_^A^ = 8.1 nm) and *r*_max_ (*r*_max_^A^ = 82 nm). For system A, we show the fitting results obtained by either fixing *r*_max_ and using *R*_0_ as a fitting parameter or by leaving both quantities as fitting parameters. For system B, we do the latter.

For system A, we obtain *R*_0_^FJC^ = 7.46 nm and *R*_0_^WLC^ = 7.2 nm for the one-parameter fits and *R*_0_^FJC^ = 5.08 nm, *r*_max_^FJC^ = 42.75 nm and *R*_0_^WLC^ = 7.2, *r*_max_^WLC^ = 63.87 nm for the two-parameter fits.

For system *B*, we obtain unphysical values (*R*_0_^FJC^ = 0.11 nm, *r*_max_^FJC^ = 0.65 nm and *R*_0_^WLC^ = 0.17, *r*_max_^WLC^ = 0.96 nm). The results do not improve if we restrict the fitting range to narrower *Q*-ranges.

## Supporting Information Available

Shear modulus in the affine network model, monomer mean-squared displacement during equilibration, and density scaling of the RMS equilibrium end-to-end distance (PDF)

## Footnotes

a In the absence of excluded volume interactions, the pressure of the system is negative and would, therefore, collapse if the volume was not kept constant.

b Here and in the following, italic is used to indicate the magnitude of the vector.

c We recall that because two cross-linkers cannot bind to each other, the minimum chain length is *n* = 1 (for two bonds)
