# Supplementary material for: Effect of Chain Polydispersity on the Elasticity of Disordered Polymer Networks
Source: Macromolecules. 2021 Apr 14;54(8):3769–79. doi: 10.1021/acs.macromol.1c00176 (PMC8154883; doi:10.1021/acs.macromol.1c00176)
Supplement: Supplementary file 1 — ma1c00176_si_001.pdf [file ma1c00176_si_001.pdf]

The effect of chain polydispersity on the elasticity of disordered polymer networks

Valerio Sorichetti,[*,†,‡,¶,§] Andrea Ninarello,[*,†,‖,⊥] José M. Ruiz-Franco,[‖,⊥] Virginie Hugouvieux,[§] Walter Kob,[¶,#] Emanuela Zaccarelli,[‖,⊥] and Lorenzo Rovigatti[*,⊥,‖]

†*These authors contributed equally*

‡*Laboratoire de Physique Théorique et Modèles Statistiques (LPTMS), CNRS, Université Paris-Saclay, F-91405 Orsay, France*

¶*Laboratoire Charles Coulomb (L2C), Univ. Montpellier, CNRS, F-34095, Montpellier, France*

§*IATE, INRAE, CIRAD, Montpellier SupAgro, Univ. Montpellier, F-34060, Montpellier, France*

‖*CNR-ISC Uos Sapienza, Piazzale A. Moro 2, IT-00185 Roma, Italy*

⊥*Department of Physics, Sapienza Università di Roma, Piazzale A. Moro 2, IT-00185 Roma, Italy*

#*Institut Universitaire de France*

E-mail: valerio.sorichetti@universite-paris-saclay.fr; andrea.ninarello@roma1.infn.it; lorenzo.rovigatti@uniroma1.it

Supporting Information

SI Shear modulus in the affine network model

Eq. 8 was obtained under the phantom network assumption, *i.e.*, that the coordinates of the vector $\mathbf{r}_\lambda = \mathbf{R}_\lambda + \mathbf{u}_\lambda$ transform according to $R_{x,\lambda} = \lambda R_{x,1}$ and $u_{x,\lambda} = u_{x,1}$. In this case, the fluctuation term is unaffected by the deformation. One can also assume, on the contrary, that the fluctuations deform affinely with the average end-to-end vector, *i.e.*, $r_{x,\lambda} = \lambda r_{x,1}$ and analogous for the other coordinates. In this case, one obtains

$$g^{\mathrm{aff}} = -\frac{TR^2}{6V}\left[\frac{ds_n(\tilde{r})}{d\tilde{r}}\frac{1}{\tilde{r}} + \frac{d^2 s_n(\tilde{r})}{d\tilde{r}^2}\right], \tag{S1}$$

which results in a different Gaussian modulus:

$$G^{\mathrm{aff,G}} = \frac{k_B T}{V}\sum_{1}^{N_s}\frac{\overline{r_i^2}}{n_i b^2} = \left\langle \frac{\overline{r^2}}{nb^2}\right\rangle k_B T\nu \equiv A^{\mathrm{aff}} k_B T\nu, \tag{S2}$$

where the sum is, as usual, taken over the N_s elastically-active strands. Under the assumption that the $\overline{r_i^2}$ are Gaussianly distributed (see Sec. AI in the main text) we have

$$\left\langle \frac{\overline{r^2}}{nb^2}\right\rangle = 1. \tag{S3}$$

From Eqs. (S3) and (S2), get the commonly reported expression[1,2]

$$G^{\mathrm{aff,G}} = k_B T\nu. \tag{S4}$$

SII Monomer mean-squared displacement during equilibration

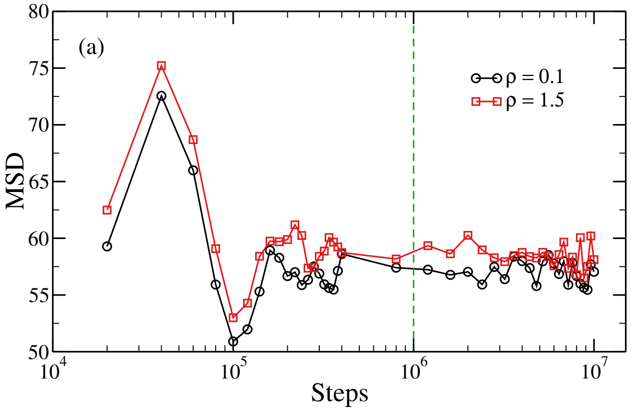

Figure S1: The mean-squared displacement of the $C = 1\%$, $\rho_{\rm init} = 0.1$ sample computed at $\rho = 0.1$ and $\rho = 1.5$. The vertical dashed line signals the equilibration time we use.

In order to verify that the system has equilibrated correctly, we measure the monomer mean-squared displacement (MSD). In Fig. S1 we report the monomer MSD of the $C = 1\%$, $\rho_{\rm init} = 0.1$ sample computed at $\rho = 0.1$ and $\rho = 1.5$. We note that the MSD quickly reaches a plateau, signaling that the oscillation modes of all the strands have equilibrated.

SIII Density scaling of RMS equilibrium end-to-end distance

We report in Fig. S2**a** for $\rho = 0.5$ the RMS equilibrium end-to-end distance of the strands, defined as $R(n) \equiv [\langle R^2(n)\rangle_n]^{1/2}$ *, where $\langle \cdot \rangle_n$ denotes the average over all the strands of length n. We note that curves for different initial densities $\rho_{\rm init}$ and crosslinker concentrations C fall on the same master curve if divided by the quantity $(\rho_{\rm init}^{\rm cl})^{1/3}$, where $\rho_{\rm init}^{\rm cl} = C\rho_{\rm init}$ is the initial crosslinker density. Since this quantity represents the inverse of the initial average distance between neighboring crosslinkers, we can conclude that the initial spatial distribution of the

*We recall that the end-to-end distance is $\mathbf{r}(t) \equiv R + \mathbf{u}(t)$, and that $\overline{r^2} = R^2 + \overline{u^2}$.

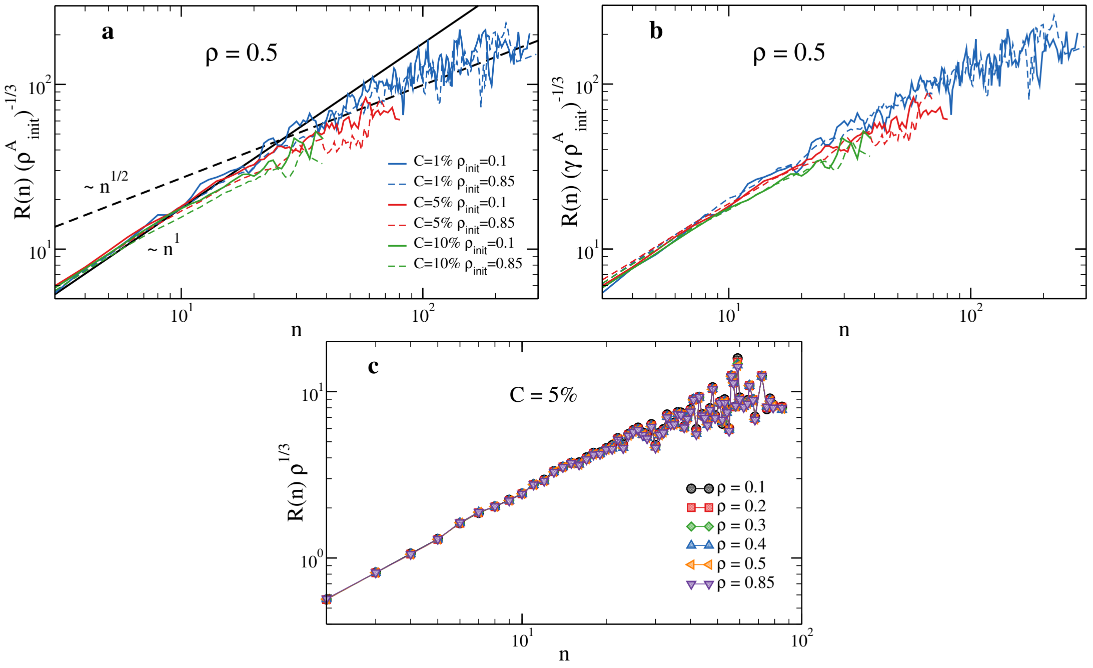

Figure S2: (**a**-**b**): RMS equilibrium end-to-end distance of the strands for $C = 1\%, 5\%, 10\%$ for $\rho_{\text{init}} = 0.1, 85$ and $\rho = 0.5$, rescaled by (**a**) the inverse of the initial average distance between neighboring crosslinkers $(\rho^A_{\text{init}})^{1/3} = (C\rho_{\text{init}})^{1/3}$ and (**b**) by $(\gamma\rho^A_{\text{init}})^{1/3}$, where $\gamma = 0.74$ for $\rho_{\text{init}} = 0.85$ and $\gamma = 1$ for $\rho_{\text{init}} = 0.1$. (**c**): Same quantity as in **a**-**b**, rescaled by $\rho^{1/3}$, for $C = 5\%$ and for different values of ρ.

crosslinkers completely controls the equilibrium end-to-end distance of the chains in the final state. An even better collapse can be obtained by using slightly different (heuristic) factors for the two values of the initial density we use here: Fig. S2**b** shows the same curves rescaled by $(\gamma\rho^A_{\text{init}})^{1/3}$, where $\gamma = 0.74$ for $\rho_{\text{init}} = 0.85$ and $\gamma = 1$ for $\rho_{\text{init}} = 0.1$. We note that the same rescaling does not apply to $[\overline{\langle r^2(n)\rangle_n}]^{1/2}$, since the fluctuation term $\overline{u^2}$ does not follow this scaling. We also report in Fig. S2**a** the scaling behavior expected for Gaussian strands, i.e., $R(n) \propto n^{1/2}$ (dashed line), and the one for stretched strands, i.e., $R(n) \propto n$ (solid line). One can see that the short chains are on average stretched, and only for larger values of n the Gaussian behavior is recovered. Finally, we remark that since the equilibrium end-to-end distances deform affinely with the network, $R(n)$ curves at different final densities ρ collapse on the same master curve when multiplied by $\rho^{1/3}$, as shown in Fig. S2**c**.

SIV Chain end-to-end distributions

In the main text we state that the non-monotonic behaviour of the shear modulus has an essentially entropic origin. Here we complement the results reported therein with two figures that strengthen this message.

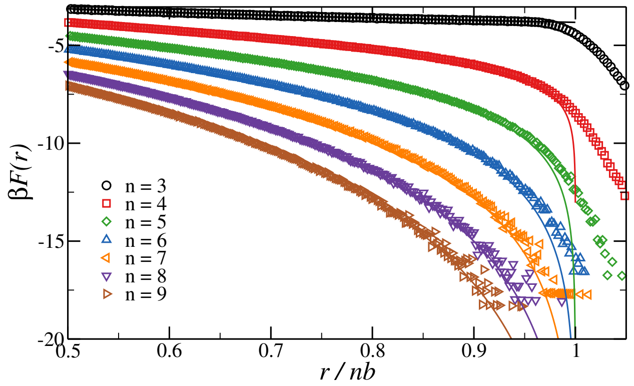

Figure S3: Free energy of chains of different n as a function of the end-to-end distance rescaled by the contour length (points) as computed in simulations and (lines) as estimated theoretically for FJCs.

Figure S3 shows the free-energy of a Kremer-Grest chain of length n and (normalised) end-to-end distance r/nb as computed in simulations (points) and estimated by using the exact FJC equation (lines). Deviations from the FJC behaviour appear always quite close to $r/nb \approx 1$ and slightly depend on n. Regardless of this value, the figure shows that the difference between the theoretical and numerical data for $r/nb < 0.95$ is always very small.

Figure S4 shows the probability distribution of the normalised chain end-to-end distance under no applied stress for all the studied systems. Every system but the three highlighted in the figure have a negligible fraction of chains that are above or close to the $r/nb \approx 0.95$ threshold, shown by the red dashed line. All in all, figures S3 and S4 show that the enthalpic contribution to the elasticity is negligible for the great majority of the systems considered here.

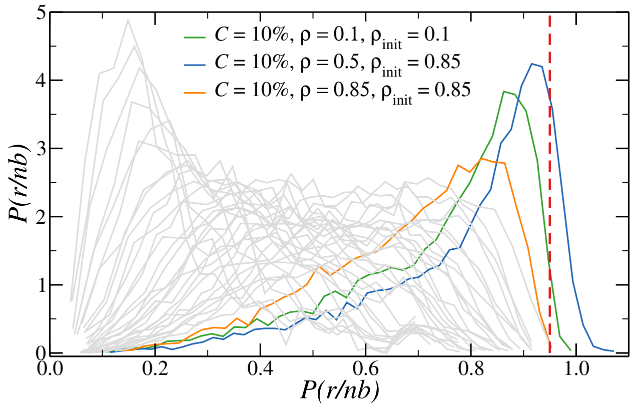

Figure S4: Probability distribution of the chain end-to-end distance rescaled by the contour length for all the investigated systems in equilibrium. The coloured curves belong to the only three systems which have a non-negligible amount of chains with $r/nb > 0.95$, and they all have $C = 10\%$. Red dashed line indicates $r/nb = 0.95$.

References

(1) Rubinstein, M.; Colby, R. H. *Polymer physics*; Oxford University Press New York, 2003.

(2) Mark, J. E. *Physical properties of polymers handbook*; Springer, 2007; Vol. 1076.
